# OsPT2, a phosphate transporter, is involved in the active uptake of selenite in rice

**DOI:** 10.1111/nph.12596

**Published:** 2013-11-11

**Authors:** Lianhe Zhang, Bin Hu, Wei Li, Ronghui Che, Kun Deng, Hua Li, Feiyan Yu, Hongqing Ling, Youjun Li, Chengcai Chu

**Affiliations:** 1 Henan University of Science and TechnologyLuoyang, 471003, China; 2 State Key Laboratory of Plant Genomics, Institute of Genetics and Developmental Biology, Chinese Academy of SciencesBeijing, 100101, China; 3 National Center for Plant Gene Research (Beijing), Institute of Genetics and Developmental Biology, Chinese Academy of SciencesBeijing, 100101, China; 4 State Key Laboratory of Plant Cell and Chromosome Engineering, Institute of Genetics and Developmental Biology, Chinese Academy of SciencesBeijing, 100101, China

**Keywords:** molecular mechanism, phosphate transporter, rice (*Oryza sativa*), selenite uptake, selenium (Se)

## Abstract

- Selenite is a predominant form of selenium (Se) available to plants, especially in anaerobic soils, but the molecular mechanism of selenite uptake by plants is not well understood.
- *ltn1*, a rice mutant previously shown to have increased phosphate (Pi) uptake, was found to exhibit higher selenite uptake than the wild-type in both concentration- and time-dependent selenite uptake assays. Respiratory inhibitors significantly inhibited selenite uptake in the wildtype and the *ltn1* mutant, indicating that selenite uptake was coupled with H^+^ and energy-dependent. Selenite uptake was greatly enhanced under Pi-starvation conditions, suggesting that Pi transporters are involved in selenite uptake.
- *OsPT2*, the most abundantly expressed Pi transporter in the roots, is also significantly up-regulated in *ltn1* and dramatically induced by Pi starvation. *OsPT2-*overexpressing and knockdown plants displayed significantly increased and decreased rates of selenite uptake, respectively, suggesting that *OsPT2* plays a crucial role in selenite uptake. Se content in rice grains also increased significantly in *OsPT2-*overexpressing plants.
- These data strongly demonstrate that selenite and Pi share similar uptake mechanisms and that *OsPT2* is involved in selenite uptake, which provides a potential strategy for breeding Se-enriched rice varieties.

## Introduction

Selenium (Se) is an essential micronutrient for humans and other animals (Schwarz & Foltz, 1957; Rotruck *et al.*, 1973). It plays important roles in antioxidant function, thyroid hormone metabolism, male fertility, and immune responses (Stadtman, 1990; Kohrle, 2000; Rayman, 2002; Stranges *et al.*, 2006). Several studies have shown that improvement of Se status can lower the risk of cancer (Clark *et al.*, 1996; Combs, 2001; Rayman, 2005). People mainly acquire Se from plant foods through the food chain, especially cereal foods (Rayman, 2012). Because of the low Se concentrations in plant foods, the average daily Se intake is insufficient to meet the requirements of human health (Williams *et al.*, 2009). Agronomic fortification was successfully performed with selenized fertilizers and resulted in an increase in Se intake, but it also increases the cost of agricultural products and brings potential environment risks (Hartikainen, 2005; Broadley *et al.*, 2006). Genetic biofortification is a new and promising strategy which may increase Se accumulation in cereal crops and reduce the need for Se fertilizers (Broadley *et al.*, 2006; Rayman, 2008). To this end, it is important to develop a comprehensive understanding of the mechanism of Se uptake in plants in order to improve the Se content in crops more efficiently.

Selenium occurs naturally as Se^2−^, Se^0^, Se_2_O_3_^2−^, SeO_3_^2−^, and SeO_4_^2−^ (Sors *et al.*, 2005). The predominant forms of Se available to plants are selenate and selenite. Redox potential and pH can greatly affect the forms of Se present in soil. Selenate is the dominant species in solution at high redox (pE + pH > 15.0). In the medium redox range (7.5 < pE + pH < 15.0), either SeO_3_^2−^ or HSeO_3_^−^ species is predominant (Elrashidi *et al.*, 1987). Sulfate transporters are involved in selenate uptake of plants (Terry *et al.*, 2000; White *et al.*, 2004; Sors *et al.*, 2005). AtSultr1;2, a high-affinity sulfate transporter, was first identified through the screening of selenate-resistant mutants in *Arabidopsis* (Shibagaki *et al.*, 2002; El Kassis *et al.*, 2007). Unlike that of selenate, the mechanism of selenite uptake in plants is not well understood. Previous studies have shown that selenite enters roots through passive diffusion (Shrift & Ulrich, 1969). This hypothesis is supported by the fact that selenite uptake is repressed only 20 or 30% by respiratory inhibitors (Arvy, 1989, 1993). However, it was shown that hydroxylamine and 2,4-dinitrophenol (DNP) inhibited selenite uptake by 72 and 86%, respectively, at pH 4.0 (Ulrich & Shrift, 1968). This discrepancy is mainly a result of the effect of pH on selenite species in the absorption solution (Zhang *et al.*, 2010). Selenous acid is a diprotic weak acid with p*K*a1 and p*K*a2 values of 2.57 and 6.60, respectively. Selenite exists as H_2_SeO_3_, SeO_3_^2−^, and HSeO_3_^−^ at different pH values (Zhang *et al.*, 2006, 2010; Zhao *et al.*, 2010). The proportions of these Se aqueous species vary greatly with pH (Zhang *et al.*, 2006, 2010; Zhao *et al.*, 2010). Selenite could be absorbed by rice roots through aquaporins as the neutral H_2_SeO_3_ species (Zhang *et al.*, 2006). Zhao *et al*. (2010) further demonstrated that a silicon (Si) influx transporter OsNIP2;1 (Lsi1), a nodulin 26-like intrinsic membrane protein (NIP) subfamily of aquaporins, is permeable to H_2_SeO_3_. However, how selenite enters roots as HSeO_3_^−^ remains unclear. Earlier studies indicated that selenite uptake was repressed by an increase in the Pi concentration (Broyer *et al.*, 1972; Hopper & Parker, 1999). Recent studies have suggested that selenite uptake is probably mediated by Pi transporters (Li *et al.*, 2008). However, there is still no convincing molecular evidence to support this hypothesis.

An ideal approach to uncovering the mechanism of selenite uptake is to identify mutants that significantly alter this uptake. Previously, the *ltn1* mutant was identified and found to be associated with leaf tip necrosis, predominantly in mature leaves, which is the result of Pi overaccumulation (Hu *et al.*, 2011). Both concentration- and time-dependent selenite uptake assays were performed to determine whether *ltn1* also has a higher uptake rate of selenite. *ltn1* was indeed found to exhibit much higher rates of selenite uptake than wild-type plants. As the transcript abundances of *OsPT1*,*OsPT2*,*OsPT4*, and *OsPT8* were significantly elevated in *ltn1* roots, it is therefore reasonable to speculate that enhanced selenite uptake in *ltn1* roots may be correlated with up-regulation of these Pi transporters. The present work demonstrated that OsPT2, a Pi transporter, is involved in selenite uptake, which provides direct evidence that this Pi transporter is also responsible for the active uptake of selenite. Se content in rice grains also increased dramatically in *OsPT2-*overexpressing plants, suggesting that this may be a suitable strategy for breeding Se-enriched rice varieties.

## Materials and Methods

### Plant materials and growth conditions

Rice (*Oryza sativa* L.) wild-type Nipponbare and its mutant (*ltn1*), wild-type Zhonghua11 and transgenic lines (Ox-1, Ox-3, Ri-2, and Ri-5) were used in this study. To evaluate selenite uptake in rice seedling, seeds were surfaced-sterilized, rinsed with deionized water, and then germinated on filter papers in an incubator at 35°C in darkness. When the radicals were 2 cm long, the seedlings were precultured in half-strength Kimura B nutrient solution (Ma *et al.*, 2001) in an environmentally controlled growth chamber, with a light : dark cycle of 16 : 8 h (24 : 18°C). The light intensity was *c*. 300 μmol m^−2^ s^−1^. The humidity was controlled at 67%. After 3 d, seedlings were transferred to full-strength nutrient solution and grown for another 12 d, and then uniform seedlings were transplanted to 18 l containers containing the same nutrient solution. The pH was adjusted to 5.5 every day with 1 mM HCl or 1 mM NaOH. The solution was replenished every 4 d. Seedlings were harvested for the Se-uptake experiment after 8 d.

### Kinetics of Se absorption

Roots of three individual plants were pretreated in Se-free solution (100 μM KH_2_PO_4_, 100 μM Ca(NO_3_)_2_ and 100 μM MgCl_2_, pH 5.5) for 0.5 h, then transferred to the absorption solution containing 5 mM MES (2-morpholinoethanesulphonic acid), 0.5 mM Ca(NO_3_)_2_ and different Se concentrations (0, 0.1, 0.2, 0.4, 0.8, 1.6, 3.2, 6.4, and 9.6 μM Na_2_SeO_3_, pH 5.5). After 3 h absorption, the roots were rinsed with an ice-cold desorption solution (5 mM MES, 0.5 mM Ca(NO_3_)_2_ and 0.5 mM K_2_SO_4_ (pH 5.5)) and then soaked in the same solution for 15 min to remove external Se. They were then blotted dry and used for Se analysis.

Similarly, roots of three individual plants were first pretreated in Se-free solution, then moved to absorption solution supplemented with 2 μM Na_2_SeO_3_ for 0.5, 1.0, 1.5, 2.0, 2.5, 3.0, 3.5, and 4.0 h. After the Se absorption, the roots were rinsed with the desorption solution and dried for Se analysis.

### Effect of respiratory inhibitors on selenite uptake

Roots of three individual plants were pretreated in Se-free solution for 30 min and then transferred to an absorption solution containing 2.0 μM Na_2_SeO_3_ with or without 1 μM carbonyl cyanide *m*-chlorophenylhydrazone (CCCP) or 0.1 mM DNP. CCCP was initially dissolved in ethanol and added to the absorption solution with a final ethanol concentration of 0.01% (v/v) (Li *et al.*, 2008). After 2 h absorption, the roots were rinsed with an ice-cold desorption solution and then immersed in the same solution for 15 min to remove external Se. Roots were blotted dry and analyzed for Se content. All reagents were purchased from Sigma.

### Examination of selenite uptake

Seedlings of wild-type and *ltn1* plants were washed thoroughly in deionized water and then transferred to normal, P-deficient or S-deficient medium. The medium was a modified version of Kimura B nutrient solution. The control was a normal nutrient solution. In the S-deficient and P-deficient solutions, KH_2_PO_4_, MgSO_4_, ZnSO_4_, and CuSO_4_ were substituted by an equimolar amount of corresponding chloride salts. After 3 d, seedlings were transferred to normal, S-deficient or P-deficient medium containing 2 μM Na_2_SeO_3_ for another 3 d, and then the roots were rinsed, dried, and analyzed for Se content.

### Vector construction and rice transformation

For overexpression vector construction, the open reading frame (ORF) of *OsPT2* was amplified and cloned into binary vector pCambia2300Actin between restriction sites *Xba*I and *Pst*I. For RNAi vector construction, a 305 bp fragment of *OsPT2* ORF was cloned in both orientations in pCambia2300Actin between restriction sites *Sal*I and *Bam*HI, separated by the first intron of GA20 oxidase of potato to form a hairpin structure (Luo *et al.*, 2005). The resulting vectors were transformed into wild-type rice (Zhonghua11) using *Agrobacterium tumefaciens*-mediated transformation (Liu *et al.*, 2006).

### Selenite uptake

Roots of three individual plants were pretreated in Se-free solution for 30 min, then transferred to the absorption solution. After absorption for 3 h, the roots were rinsed with an ice-cold desorption solution, then soaked in it for 15 min to clean up the external Se, and then blotted dry before separating roots and shoots for Se analysis.

### *OsPT2* RNA *in situ* hybridization

RNA *in situ* hybridization was performed as previously described (Li *et al.*, 2011). The primers used for probe amplification are GCACAAACTTCCTCGGTATG (ISPTZ-1F, sense probe) and AAGAGAAACCCCACAAATCC (ISPTZ-1R, antisense probe). Digoxigenin-labeled RNA probes were prepared using a DIG Northern Starter Kit (catalogue no. 2039672; Roche) according to the manufacturer's instructions. The hybridization signals were observed using bright field imaging with a microscope (Olympus BX51, Tokyo, Japan) and photographed with a Micro Color CCD camera (DVC Co., Austin, TX, USA).

### Field experiment

Wild-type rice (Zhonghua11) and transgenic lines (Ox-1, Ox-3, Ri-2, and Ri-5) were planted in the field of the experimental station of the Institute of Genetics and Developmental Biology (IGDB), Chinese Academy of Sciences (CAS), in Lingshui County of Hainan Province, China. The area of the plot for wildtype rice and each transgenic line was 10 m^2^, with three replicates. The soil type is clay with pH 5.2. The concentrations of organic matter, available N, available P and available K were 19 620, 123.67, 32.64, and 137.23 mg kg^−1^, respectively. The concentrations of total Se and available Se in the soil were 0.18 mg kg^−1^ and 5.23 μg kg^−1^, respectively. The cultural management practices were the same for wild-type and transgenic rice. Upon ripening, the rice was harvested and the grains were dehusked by hand. The Se content was determined in brown rice grains.

### Analysis of total Se

All glass vessels were initially soaked overnight in 10% HCl to eliminate Se contamination, then rinsed in double deionized water (> 18 MΩ) and oven-dried. Dried and homogenized samples (*c*. 100 mg) were weighed and put into 100 ml digestion tubes. Then 5 ml of an acid mixture of 4 ml HNO_3_ and 1 ml HClO_4_ were added. Samples were digested overnight at 25°C, then completely digested at 150°C in a digestion oven. After cooling, the digests were diluted with double deionized water to a final volume of 25 ml. Total Se in the digested samples was determined by inductively coupled plasma mass spectrometry (ICP-MS). All chemical reagents used were of a high-purity grade. A standard tea material (GSV-4, 0.072 mg Se kg^−1^, GBW07605; The Institute of Geophysical and Geochemical Exploration, Langfang City, China) and a blank were simultaneously digested with the test samples for quality control, with an average Se recovery of between 89 and 93% (Zhang *et al.*, 2006).

### Statistical analysis

Analysis of variance was performed using SPSS 13.0 for Windows (SPSS Inc., Chicago, IL, USA) to determine the significance of any differences between control and treatments. Statistical differences were assessed by Student's *t*-test. All data shown were the means of at least three samples.

## Results

### Selenite uptake is significantly increased in the *ltn1* mutant

**Figure 1:**
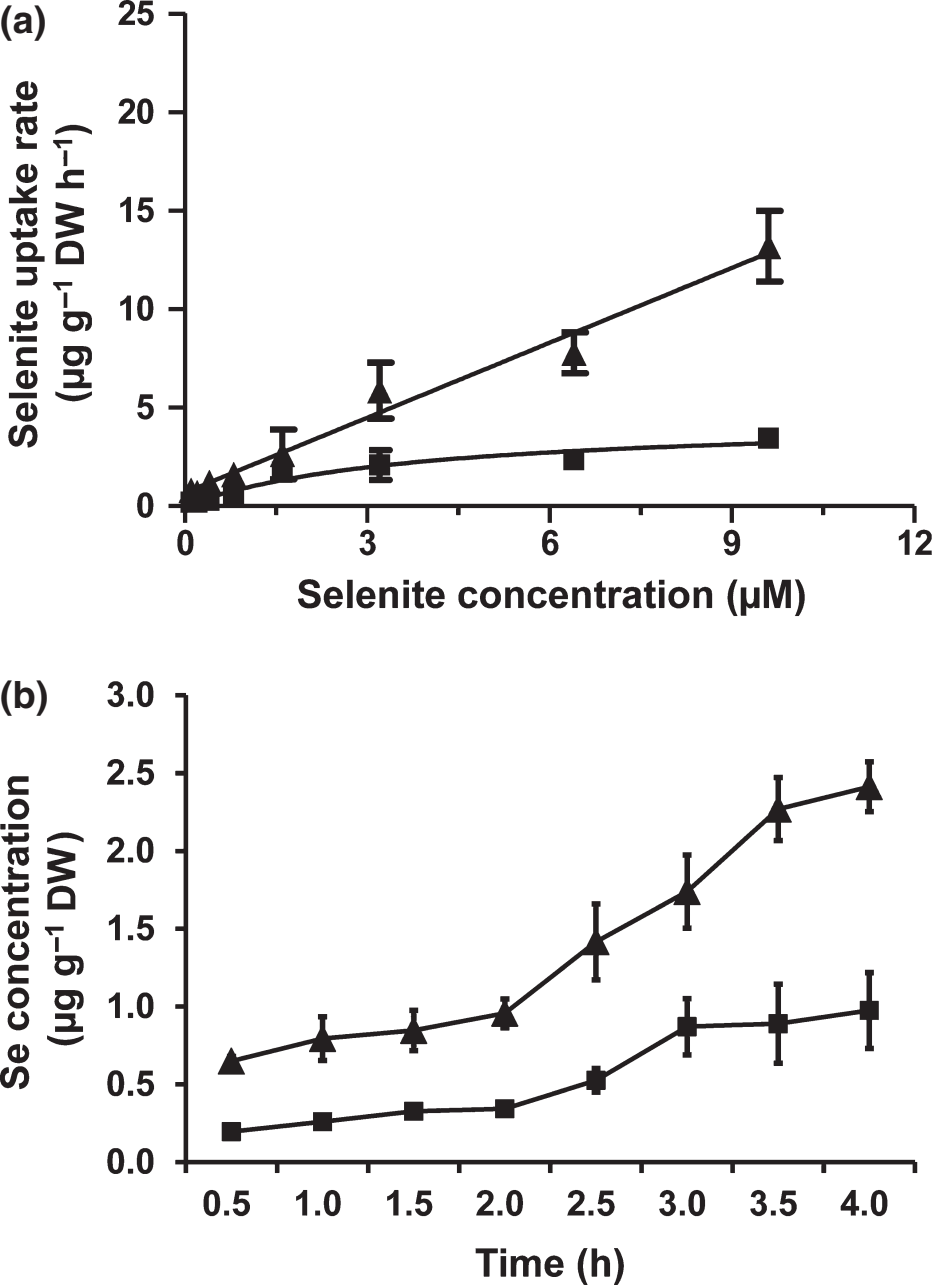
Difference in concentration- (a) and time-dependent (b) selenite uptake by roots of Nipponbare (*Oryza sativa*; squares) and its mutant *ltn1* (triangles). Error bars indicate mean values ± SD (*n* = 5).

Both concentration- and time-dependent selenite uptake experiments were performed to evaluate whether *ltn1* has a higher rate of uptake of selenite than the wild-type. Concentration-dependent kinetics suggested that selenite uptake by *ltn1* increased in proportion to the Se concentration in the absorption solution (Fig.1a). A linear equation was fitted to the data with regression coefficients of 0.99. Selenite uptake by *ltn1* became significantly higher than the wild-type as Se concentrations increased; however, selenite uptake by the wild-type followed saturation kinetics as Se concentrations increased. The data fitted a Michaelis–Menten saturation curve (*R*^2^ = 0.98). *V*_max_ (maximum enzymatic reaction rate) was 4.45 μmol kg^−1^ DW h^−1^, and *K*_m_ (substrate concentration at which the reaction rate is half maximum) was 3.77 μmol kg^−1^ DW. Time-dependent selenite uptake showed that *ltn1* had significantly higher Se concentrations than the wild-type at all Se-treated time-points (Fig.1b). After 3 h exposure, Se concentrations in wild-type roots almost reached a plateau, whereas Se concentrations in *ltn1* kept increasing with extending Se treatment. Given that *ltn1* was characterized as a Pi overaccumulation mutant, it was reasonable to speculate that selenite uptake might be associated with the Pi uptake pathway.

### Selenite uptake is enhanced by Pi starvation

**Figure 2:**
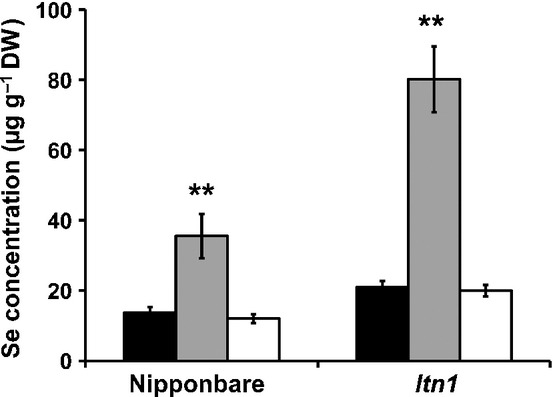
Effects of phosphorus (P) and sulfur (S) starvation on selenium (Se) concentration in roots of Nipponbare (the wild-type, *Oryza sativa*) and its mutant *ltn1*. Error bars indicate mean values ± SD (*n* = 5). Asterisks indicate the significant differences between the wild-type and *ltn1* as determined by Student's *t*-test: **, *P *< 0.01. Control, black bars; no P, gray bars; no S, white bars.

To investigate the effects of P or S starvation on selenite uptake, wild-type and *ltn1* rice seedlings were grown in normal, P-deficient, or S-deficient medium. After 3 d, seedlings were then transferred, respectively, to normal, P-, or S-deficient medium containing 2 μM Na_2_SeO_3_ for another 3 d and the Se content was determined. The results showed that the Se content in the roots of wild-type plants and *ltn1* mutants in P-deficient medium was significantly higher than that of the control, but S starvation had no effect on the Se content of either wild-type plants or *ltn1* mutants (Fig.2). Under P-starvation conditions, the concentrations of Se in roots of the wild-type and *ltn1* mutants were increased 2.58- and 3.81-fold relative to the control, respectively. These results showed that the selenite uptake capacity of wild-type and *ltn1* plants was significantly increased under P-deficient conditions, indicated that Pi deficiency dramatically promotes selenite uptake.

### Selenite uptake was mediated by symport with H^+^

**Figure 3:**
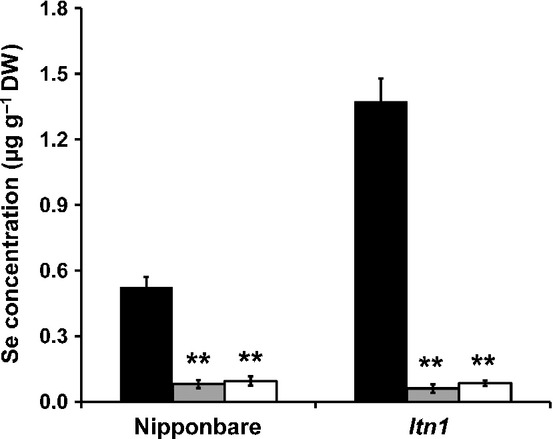
Effects of carbonyl cyanide *m*-chlorophenylhydrazone (CCCP) and 2,4-dinitrophenol (DNP) on selenite uptake by roots of Nipponbare (the wild-type, *Oryza sativa*) and *ltn1*. Error bars indicate mean values ± SD (*n* = 5). Asterisks indicate the significant differences between the wild-type and *ltn1* as determined by Student's *t*-test: **, *P* < 0.01. Control, black bars; CCCP, gray bars; DNP, white bars.

To determine whether selenite uptake was mediated by symport of H^+^, selenite uptake of wild-type and *ltn1* plants was measured after the roots had been exposed to 2 μM Na_2_SeO_3_ absorption solutions containing 1 μM CCCP or 20 μM DNP for 2 h. The control was supplied with the same absorption solutions without CCCP or DNP. Selenite uptake of both wild-type and *ltn1* plants was significantly lower than in controls after addition of 1 μM CCCP or 20 μM DNP to the absorption solutions, and the rate of selenite uptake of *ltn1* plants was reduced to the same value as the wild-type (Fig.3). Both CCCP and DNP are typical protonophores, which allow protons to freely transverse the membrane and inhibit anion uptake by depolarizing the electrical potential across the plasma membrane (Shioi & Taylor, 1984). These results indicated that selenite uptake was energy-dependent and mediated by symport of H^+^ and selenite anion, which is consistent with Pi uptake (Pao *et al.*, 1998).

### *OsPT2* was expressed most abundantly under Pi-deficient conditions

**Figure 4:**
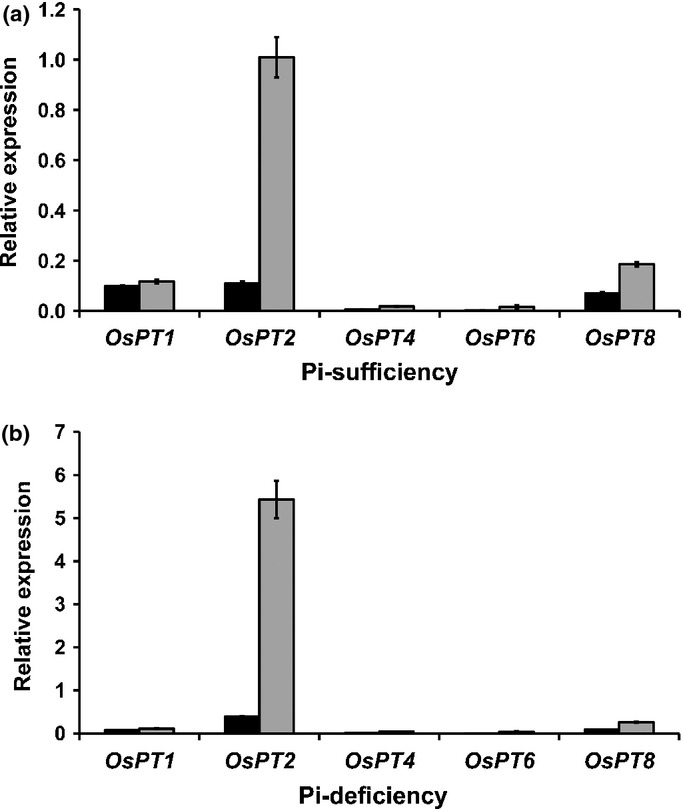
Effect of phosphorus (P) on gene expression of Pi transporters in roots of Nipponbare (the wild-type, *Oryza sativa*; black bars) and *ltn1* (gray bars) under Pi-sufficient (a) and Pi-deficient (b) conditions. Error bars indicate mean values ± SD (*n* = 3).

To examine the expression of Pi transporters, the wild-type and *ltn1* plants were exposed to Pi-sufficient and Pi-deficient medium and the expression of the Pi transporters was determined by real-time quantitative reverse transcription polymerase chain reaction (qRT-PCR). The results showed that the expression of *OsPT1*,*OsPT2*,*OsPT4*,*OsPT6*, and *OsPT8* was substantially up-regulated in *ltn1* relative to the wild-type under Pi-sufficient conditions, as in previous results (Fig.4a). Under Pi-deficient conditions, the expression of *OsPT1*,*OsPT2*,*OsPT4*,*OsPT6*, and *OsPT8* was substantially up-regulated in both the wild-type and *ltn1* (Fig.4b). The expression of *OsPT2* was the most abundant and was far more highly expressed than other Pi transporters. These results indicate that *OsPT2* probably plays a major role in either Pi or selenite uptake in rice.

### Selenite uptake and selenium accumulation were correlated with *OsPT2* expression

**Figure 5:**
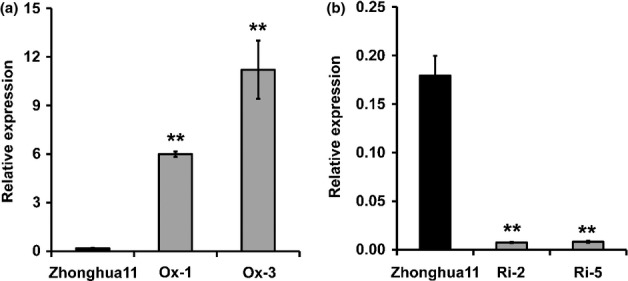
Expression of the *OsPT2* gene in Zhonghua11 (*Oryza sativa*) and *OsPT2* transgenic lines. Ox-1 and Ox-3 are two *OsPT2-*overexpressing lines; Ri-2 and Ri-5 are two *OsPT2-*RNAi lines. Error bars indicate mean values ± SD (*n* = 3). Asterisks indicate the significant differences between Zhonghua11 and *OsPT2* transgenic lines as determined by Student's *t*-test: **, *P* < 0.01.

To further confirm the involvement of OsPT2 in selenite uptake, transgenic rice plants with overexpressing *OsPT2* and knockdown *OsPT2* by RNAi were generated. The results showed that the expression of *OsPT2* was significantly higher in the overexpression lines but dramatically repressed in the RNAi lines (Fig.5a,b).

**Figure 6:**
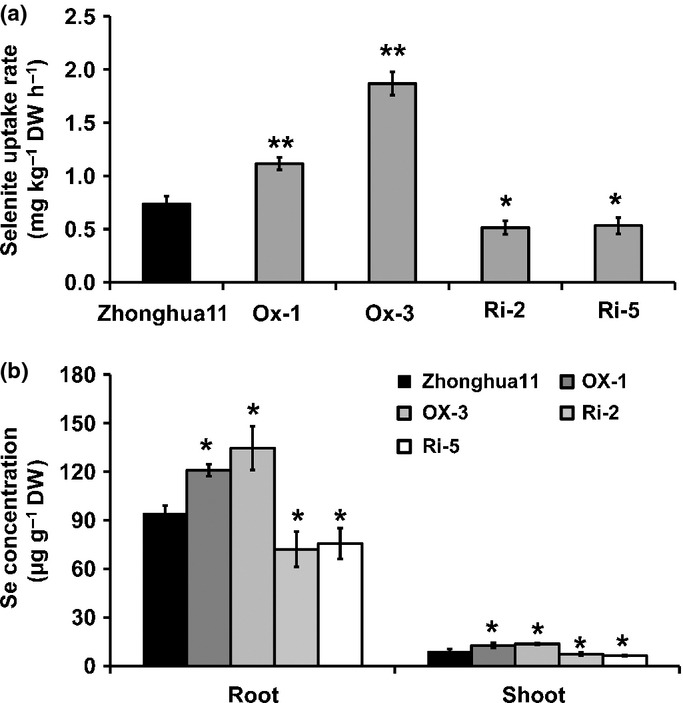
(a) Difference in selenite uptake by roots of Zhonghua11 (*Oryza sativa*) and *OsPT2* transgenic lines. (b) Difference in selenium (Se) concentration in roots and shoots of Zhonghua11 and transgenic lines. Ox-1 and Ox-3 are two *OsPT2-*overexpressing lines; Ri-2 and Ri-5 are two *OsPT2-*RNAi lines. Error bars indicate mean values ± SD (*n* = 3). Asterisks indicate the significant differences between Zhonghua11 and *OsPT2* transgenic lines as determined by Student's *t*-test: *, *P* < 0.05; **, *P* < 0.01.

To investigate the relationship between selenite uptake and *OsPT2* expression, the selenite uptake rate of the wild-type, *OsPT2*-overexpressing lines, and *OsPT2* RNAi lines were compared in the presence of 2 μM Na_2_SeO_3_. After 3 h of incubation, the uptake rate of the two overexpression lines was significantly higher and that of the two RNAi lines was significantly lower than the wild-type (Fig.6a). After 4 d of incubation, Se content in these two overexpression lines was significantly higher than the wild-type in both roots and shoots. By contrast, the Se content of the RNAi lines was significantly lower than in the wild-type (Fig.6b). These results further demonstrated that OsPT2 played a crucial role in selenite uptake.

### *OsPT2* was predominantly expressed in epidermal cells of primary roots

**Figure 7:**
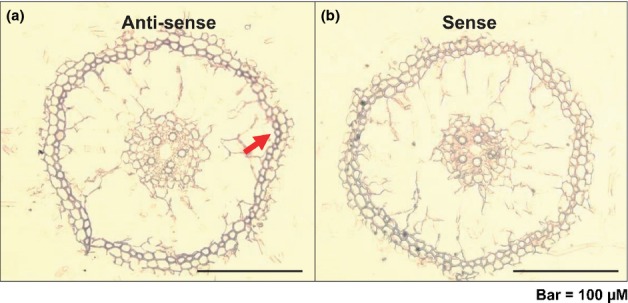
(a) *OsPT2* tissue location in young root detected by mRNA *in situ* hybridization. The *OsPT2* signal was detected in the epidermal tissue (indicated by a red arrow) of the primary roots of Zhonghua11 (*Oryza sativa*). (b) Negative control preparation made with an *OsPT2* sense probe.

Previous studies have shown that OsPT2 can mediate Pi uptake from the external solution (Ai *et al.*, 2009). It was therefore postulated that *OsPT2* may be expressed in the epidermal cells of the primary root under P-deprived conditions. To confirm this hypothesis, RNA *in situ* hybridization was performed. Results showed that *OsPT2* was highly expressed in the epidermal tissue of the primary roots with an *OsPT2* antisense probe (Fig.7a). By contrast, hybridization with an *OsPT2* sense probe showed no signal (Fig.7b). This location of the tissue strongly supported the conclusion that OsPT2 could mediate Pi uptake by the roots.

### Se content was increased in rice grains of *OsPT2*-overexpressing plants

**Figure 8:**
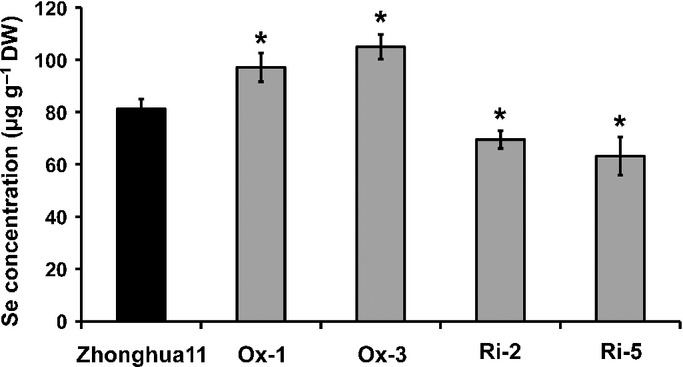
Selenium (Se) concentration in rice grains of Zhonghua11 (*Oryza sativa*) and *OsPT2* transgenic lines. Ox-1 and Ox-3 are two *OsPT2-*overexpressing lines; Ri-2 and Ri-5 are two *OsPT2-*RNAi lines. Error bars indicate mean values ± SD (*n* = 3). Asterisks indicate the significant differences between Zhonghua11 (*Oryza sativa*) and *OsPT2* transgenic lines as determined by Student's *t*-test: *, *P *< 0.05.

To investigate the effect of *OsPT2* gene expression on Se content of rice grains, wild-type and transgenic rice plants were planted in a field and given the same cultural management. After harvest, the Se content of the grains was determined. The results showed that the Se content in rice grains of *OsPT2*-overexpressing plants (Ox-1 and Ox-3) was significantly higher than in the wild-type (Fig.8). Consistently, the Se content was significantly lower than the wild-type in the grains of *OsPT2* RNAi lines (Ri-2 and Ri-5) (Fig.8) These results further demonstrated that Se content in seeds can be improved by enhanced Se uptake.

## Discussion

### Active uptake of selenite was mediated by Pi transporters

In the present study, it was shown that *ltn1* exhibited a much higher selenite uptake rate than the wild-type. The *ltn1* mutant was previously identified with Pi overaccumulation in the leaves (Hu *et al.*, 2011). It has been strongly suggested that selenite uptake has a direct relationship with Pi uptake and may be mediated by Pi transporters. CCCP and DNP are typical protonophores, which allow protons to freely transverse the membrane and collapse the proton motive force, which results in the inhibition of anion uptake (Shioi & Taylor, 1984). Both CCCP and DNP significantly inhibited selenite uptake in wild-type and *ltn1* plants, suggesting that the selenite uptake process is energy-dependent and involves symport of H^+^ and selenite anion. Previous studies have demonstrated that Pi uptake is strongly depressed by CCCP and DNP and mediated by H^+^/H_2_PO_4_^−^ symport across the plasma membrane (Ullrich-Eberius *et al.*, 1981; Leggewie *et al.*, 1997; Daram *et al.*, 1998; Liu *et al.*, 1998; Raghothama, 1999). Selenite uptake was therefore postulated to be closely related to the H^+^/H_2_PO_4_^−^ symporter.

### OsPT2 is responsible for selenite uptake

A large number of the genes encoding Pi transporters have been identified in different plant species and are generally classified into Pht1, Pht2, Pht3, and PT gene families (Rausch & Bucher, 2002). The Pht1 family plays a crucial role in both Pi uptake from soil and translocation from roots to shoots. There are 13 members of the Pht1 family in the rice genome, of which 10 are expressed in roots; the transcripts of *OsPT12* and *OsPT13* were not detected in roots and the mRNA of *OsPT7* was barely detectable (Paszkowski *et al.*, 2002). Expression analysis revealed transcripts of *OsPT1*,*OsPT2*,*OsPT4*, and *OsPT8* to be significantly up-regulated in *ltn1* roots relative to the wild-type, with *OsPT2* showing the highest expression. Under Pi-deficient conditions, the transcripts of *OsPT1*,*OsPT2*,*OsPT4*, and *OsPT8* were significantly up-regulated in both the wild-type and *ltn1* roots, and the expression of *OsPT2* was the most abundant. Selenite uptake assays showed that *ltn1* exhibits greater selenite uptake than the wild-type at different Se concentrations and time-points during Se treatment. Moreover, selenite uptake was much more pronounced in both wild-type and *ltn1* plants under Pi-starvation conditions than under normal conditions, which can activate Pi transporters to increase Pi uptake. Therefore, it could be concluded that Pi transport is involved in selenite uptake. Among the Pi transporters, *OsPT2* was found to be the most abundantly expressed one and most significantly up-regulated in *ltn1*, and was also more dramatically induced by Pi starvation. Therefore, it is most likely that enhanced selenite uptake in both the wildtype and *ltn1* roots is mainly correlated with up-regulation of *OsPT2* gene expression. The rate of selenite uptake was significantly higher in *OsPT2* overexpression lines than in the wild-type and significantly lower in RNAi lines, strongly suggesting that OsPT2 was involved in selenite uptake.

### Putative selenite-H^+^ symport process in the plant cell membrane

Plant Pht1 transporters belong to the major facilitator superfamily (MFS) transporters, which transduce electrochemical energy stored in the proton gradients into substrate concentration gradients (Saier *et al.*, 1999; Kaback *et al.*, 2001). The bacterial MFS transporters have 12 transmembrane domains, and both the N- and C-terminal halves surround a central hydrophilic cavity containing the substrate-binding sites determining specificity (Maiden *et al.*, 1987; Pao *et al.*, 1998; Hirai *et al.*, 2002). Plant Pi transporters are predicted to have 12 transmembrane domains (TMs), containing two partially duplicated subdomains of six TM segments (Saier, 2000; Lagerstedt *et al.*, 2004). To date, the process of phosphate-H^+^ symport in the membrane is still unclear. The putative mechanism of proton and Pi symport through a Pht1 transporter is consistent with the mechanism of proton and glycerol-3-phosphate symport in *Escherichia coli* (Abramson *et al.*, 2003; Huang *et al.*, 2003). Therefore, a possible symport process of proton and selenite through a phosphate-H^+^ symporter might be involved in protonation of OsPT2, with hydrophilic pores open outside and binding of the selenite anions, and then conformational change leading to opening of the pores inside, the release of selenite, OsPT2 deprotonation, and a return to the outward face conformation.

The process of phosphate-H^+^ symport was initially involved in Pi recognition. The specificity to Pi in substrate-binding sites in the transporter is determined by a few key amino acid residues. Arg45 and Arg269 are located at the proposed substrate-binding site and often participate in Pi recognition in proteins by forming hydrogen bonds with its oxygen atoms in glycerol-3-phosphate transporter from *Escherichia coli* (Huang *et al.*, 2003). However, the Pi recognition site in plant Pi transporters is unclear. Previous studies have shown that a number of plant transporters do not exhibit high specificity for substrates. For example, an Arabidopsis sulfate transporter, Sultr1;2, identified from screening for mutants resistant to selenate, can also transport selenate, because of the structural and chemical similarity to sulfate (Shibagaki *et al.*, 2002; El Kassis *et al.*, 2007). A silicon influx transporter, OsNIP2;1, is permeable to arsenite, methylated arsenic, and selenite in rice roots (Ma *et al.*, 2008; Zhao *et al.*, 2010). In our work, OsPT2 was found to transport selenite as well as Pi, indicating that it does not have a highly specific binding site for Pi.

### OsPT2 participates in selenite uptake but not root-to-shoot translocation

OsPT2's main role may be in Pi translocation, and up-regulation of *OsPT2* may elevate Pi in rice shoots and result in Pi toxicity under normal conditions (Ai *et al.*, 2009). However, the selenite that was taken up was poorly translocated to shoots and retained in the roots of plants overexpressing *OsPT2*. Previous studies have shown that selenite does not normally accumulate in the roots, and most of the Se in selenite-fed plants was converted to selenate or an unknown compound, postulated to be a selenotrisulfide (Asher *et al.*, 1977). When treated with selenite for 1 h, *Astragalus crotalariae* contained *c*. 4% selenite, 2% selenate, and 92% as a neutral or basic form, most probably MeSeCys (Shrift & Ulrich, 1969). *Brassica juncea* treated with selenite showed 4.3% selenite, 51.2% selenomethionine Se-oxide hydrate, 1.2% S-(methylseleno) cysteine, and 34.2% selenomethionine accumulation in the shoots (Kahakachchi *et al.*, 2004). In selenite-treated plants, selenomethionine, selenomethionine Se-oxide, Se-methyl-selenocysteine, and several other unidentified Se species were detected in the root extracts and xylem sap with limited translocation to shoots (Li *et al.*, 2008). These results suggest that the major portion of selenite was readily reduced to organic Se compounds such as selenomethionine in roots. This explains why OsPT2 was not involved in selenite translocation from roots to shoots. However, the Se content was higher in the shoots of *OsPT2-*overexpressing plants than in the wild-type. This was predominantly the result of involvement of OsPT2 in selenite uptake and the resulting higher rate of Se accumulation in roots. Consequently, more selenite was speculated to be converted to organic Se and then transported to shoots, which resulted in greater Se accumulation in shoots. Therefore, increasing selenite uptake is a prerequisite to Se accumulation in shoots.

*OsPT2* is predominantly expressed in roots. Under P-deprived conditions, it was strongly induced and expressed in the stele of primary roots and lateral roots, but not in epidermal or cortical cells (Ai *et al.*, 2009). However, selenite was readily converted to other organic forms in epidermal and cortical cells before transportation to the stele. This raises the issue why OsPT2 could increase selenite uptake in roots if it could not transport organic Se. In this study, *OsPT2* was also found to be highly expressed in the epidermal tissues of the primary roots, further supporting the idea of involvement of OsPT2 in selenite uptake. One reasonable explanation is that *OsPT2* might also be expressed in root epidermal and cortical cells, depending on the developmental stages of root formation.

### Overexpression of *OsPT2* can improve Se content in rice grains

A global survey of Se in rice showed that Se concentrations in major rice-producing and rice-consuming countries are low and *c*. 75% of the grains from the production and export pools would fail to provide 70% of daily recommended Se intake (Williams *et al.*, 2009). It is well documented that spraying foliage with Se was an effective way to increase Se content in rice grains and is widely used in agriculture (Cao *et al.*, 2001; Hu *et al.*, 2002). However, this practice is often limited by weather factors such as wind and rain. Moreover, the application of Se to foliage may pose some environmental risks. In the present study, *OsPT2*-overexpressing plants showed significantly higher Se content in rice grains than the wild-type, suggesting that genetic biofortification of Se in rice grains is a feasible means of increasing Se uptake, which may replace foliage spray or application of Se fertilizers in soils. When selenite is absorbed by plants overexpressing *OsPT2*, most of that Se is accumulated in the roots. The next target might be to facilitate the root-to-shoot movement of Se to further improve Se content in rice grains. It is therefore very important to identify transporters responsible for organic Se translocation from roots to shoots.

In conclusion, the Pi transporter OsPT2 is the first transporter identified in plants that is responsible for the active uptake of selenite. Overexpression of *OsPT2* could significantly increase selenite uptake and Se accumulation in both shoots and roots, thus resulting in a higher Se content in rice grains, which could be a potential strategy for breeding Se-enriched rice varieties.
